# Cyclic testing reliability analysis on a novel light-curable bone fixation technique

**DOI:** 10.3389/fbioe.2025.1515319

**Published:** 2025-07-01

**Authors:** Paula M. N. Cameron, Daniel J. Hutchinson, Micheal Malkoch, Peter Varga, Peter Schwarzenberg

**Affiliations:** ^1^ AO Research Institute Davos, Davos, Switzerland; ^2^ Department of Fibre and Polymer Technology, KTH Royal Institute of Technology, Stockholm, Sweden

**Keywords:** customizable osteosynthesis, patient-specific osteosynthesis, cyclic testing, fatigue strength, failure analyses

## Abstract

Metal fixation is currently the standard of care for treating bone fractures surgically, as it provides ample stability to the healing bone. However, metal components have been associated with soft tissue adhesions and are generally not patient specific. A novel light-curable bone fixation method, called AdhFix, overcomes these disadvantages by allowing for *in situ* customizability and demonstrating a lack of soft tissue adhesions. Previous studies on this fixation technique have demonstrated the maximum bending and torsional moments in monotonic failure tests in dry conditions. However, this fixation has yet to be tested cyclically in a more physiological environment, which would represent an important step to assessing the clinical efficacy of this technology. This study aims to test the novel fixation method cyclically at relevant force levels in a controlled near-physiological environment. Midshaft osteotomies were performed on ovine proximal phalanges which were then fixated with the AdhFix osteosynthesis technique. The constructs were tested cyclically in four-point bending for 12,600 cycles, representing 6 weeks of rehabilitation, or until failure, while submerged in Ringer solution at 37°C. The samples were divided into four groups, each tested with a different peak force. The peak forces were based on safety factors (Group 1: 100x, Group 2: 150x, Group 3: 175x, Group 4: 250x) of a physiological bending moment present in a human proximal phalanx osteosynthesis during rehabilitation exercises, determined in a previous study. All samples survived at the lowest peak moment (Group 1), whereas all failed at the highest peak force (Group 4). Kaplan-Meier curves represented the survival probability as a function of the number of cycles for each group, and a log-rank test revealed that the survival curves were significantly different (p < 0.001). The difference in patch height between the failures and survivors was not statistically significant (p = 0.113), but the final cycle displacement amplitude was statistically different (p < 0.001). This study found that this novel osteosynthesis method can survive a clinically relevant number of cycles at a force level 100× the bending loads involved in typical non-weight-bearing rehabilitation exercises. Further studies are needed to confirm safety for other conditions.

## 1 Introduction

Hand fractures are among the most common type of upper extremity bone fracture (Dominguez-Prado et al., 2022; Karl et al., 2015; Moura et al., 2022). The treatment outcomes for hand fractures, particularly phalangeal fractures, are often poor, with a high prevalence of joint stiffness and decreased range of motion (Hardy, 2004; Isaksson, 2012; von Kieseritzky et al., 2017; Pag et al., 1998). Early mobilization is a key factor in improving range of motion following a fracture (Hardy, 2004; Onishi et al., 2015). To allow for early rehabilitation and mobilization, open reduction and internal fixation (ORIF) is commonly used, especially in the case of comminuted fractures or fractures in multiple fingers (Hardy, 2004; Carpenter and Rohde, 2013; Cheah and Yao, 2016). The current standard fixation techniques for phalangeal fractures requiring internal fixation include metal plates, screws, and wires (Logters et al., 2018). While these implants provide significant strength and stability to the healing bone, they are not without disadvantages. In reference to metal plates specifically, they can lead to soft tissue adhesion and damage requiring implant removal in an additional surgery. Moreover, they are generally manufactured in a set of standard sizes meaning that they are not customizable to the size and shape of each patient’s bones (Logters et al., 2018; Kim et al., 2020).

This study investigates the use of a biocompatible light-curable composite mixture (Bonevolent™ AdhFix, Biomedical Bonding AB, Stockholm, Sweden) that is coupled with cortical screws to create a customizable fracture fixation patch. At the time of publication, AdhFix was not a clinically available certified medical device and was non-biodegradable. The main benefits of AdhFix are its *in situ* customizability and the composite’s lack of tissue adhesions as demonstrated in previous studies (Kieseritzky et al., 2020; Granskog et al., 2018; Hutchinson et al., 2021). Previous biomechanical studies on this fixation technique have focused on determining the maximum bending moment with monotonic tests in dry conditions and on evaluating the inter- and intra-operator variability when constructing these patches (Colding-Rasmussen et al., 2023; Schwarzenberg et al., 2023).

Hand rehabilitation following a fracture consists of various exercises that initially do not load the fractured bone but serve to maintain tendon motion and decrease joint stiffness (Hardy, 2004). Finger-to-palm flexion is a common exercise a clinician could prescribe to facilitate rehabilitation (Miller et al., 2016). A recent study aimed to determine the internal loads experienced in a proximal phalanx fracture osteosynthesis using experiments and computational modelling (Schwarzenberg et al., 2024). That study found that the osteosynthesis experienced internal moments of 6.78 ± 1.62 Nmm during finger-to-palm flexion exercises (Schwarzenberg et al., 2024). This provides an estimate of the loads that an osteosynthesis device needs to be able to withstand in a human proximal phalanx. Beyond supporting the physiological loading, the osteosynthesis must support this load cyclically throughout the entire rehabilitation period.

In the development of a new osteosynthesis technique, such as AdhFix, it is crucial to understand the behavior of the implant when loaded cyclically (Kim et al., 2020). Furthermore, when conducting cyclic testing, it is important to consider how many cycles constitute a clinically relevant number. In the context of hand rehabilitation, bone healing takes at least 6 weeks (Hardy, 2004). Patients whose fracture was repaired with an internal fixation are generally able to start rehabilitation immediately, but the first six weeks should be constrained to exercises without any load (Miller et al., 2016). Estimating the number of cycles performed during hand rehabilitation requires making a few assumptions about the number of exercises and the number of repetitions of each exercise performed each day. In one study, five exercises were prescribed, to be repeated 10 times each, six times per day, totaling 300 cycles per day (Miller et al., 2016). Another method to estimate the number of cycles would be to assume that an ergotherapy session lasts approximately 25 min, and that a patient would perform the exercises at a frequency of approximately 0.2 Hz, also totaling to 300 cycles per day (Pastor et al., 2024). In either case, across 6 weeks of healing, we can expect approximately 12,600 cycles of rehabilitation exercises to be performed. This indicates the minimum number of cycles the osteosynthesis should survive and guided this study’s methods.

The aim of this study was to test the AdhFix patches applied to ovine proximal phalanx bones cyclically in controlled physiological-mimicking conditions to understand the fatigue behavior of this novel osteosynthesis technique. More specifically, the objective was to find the lower and upper load limits ensuring survival and causing failure, respectively, thereby gaining an understanding of the forces at which these patches are not expected to experience a fatigue failure.

## 2 Materials and methods

### 2.1 Specimen information and sample preparation

**FIGURE 1:**
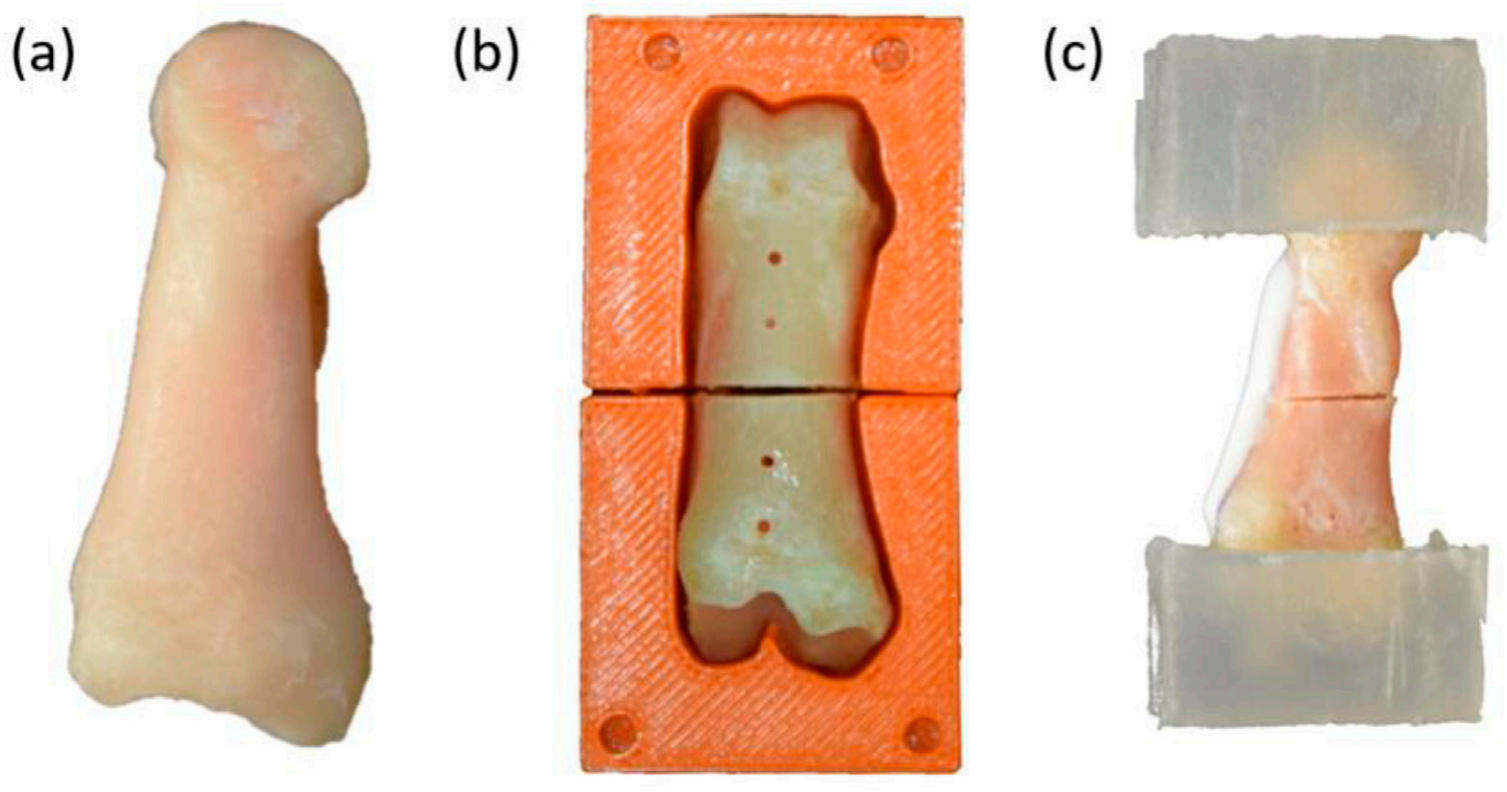
Sample preparation process. **(a)** Lateral view of a dissected ovine phalanx bone. **(b)** Anterior view of the bone positioned in the cutting guide after the creation of the osteotomy and the drilling of the four pilot holes in the dorsal side. **(c)** Lateral view with the bone embedded in PMMA blocks, with AdhFix osteosynthesis applied. The composite is visible as a white patch on the dorsal side of the bone.

Forty proximal phalanges were dissected from skeletally mature female Swiss alpine sheep (age: 3.7 ± 1.1 years, weight: 73.6 ± 7.3 kg) for use in this study. No animals were euthanized for the purpose of this study as the sheep used were from other studies for which the ethical approval was previously obtained. The bones were dissected, stripped of all soft tissue including the periosteum, and wrapped in gauze soaked with Ringer’s solution, an electrolyte-rich fluid commonly used to maintain hydration and physiological conditions in bone samples (Figure 1a). Next, the phalanges were scanned with a high-resolution peripheral quantitative CT (HR-pQCT) scanner (XtremeCT II, Scanco Medical AG, Brüttisellen, Switzerland) with an X-ray tube voltage of 68 kVp, an X-ray tube current of 1,465 μA, and an isotropic voxel size of 90 µm. Custom specimen-specific osteotomy guides were created from the CT scans using image segmentation and Boolean operations in Amira 3D (version 2021.1, Thermo Fisher Scientific) and 3D printed on a Stratasys F170 3D Printer (Stratasys Ltd., Rehovot, Israel), using a previously established protocol (Schwarzenberg et al., 2023). The cutting guides were designed with a cutting slot, to perform a reduced osteotomy at midshaft, and four holes placed longitudinally, to drill the pilot holes into the dorsal side of the bone samples as demonstrated in Figure 1b. Two pilot holes were placed on either side of the cutting slot spaced 5 mm apart and 5 mm from the cutting slot.

The 40 proximal phalanges were divided into four testing groups with ten samples in each group. Each group was tested cyclically with a different peak force. The peak force levels were initially determined from pilot testing. Then, each selected peak force was rounded to represent a different safety factor of the physiological bending moment measured in a previous human cadaver proximal phalanx osteosynthesis (Schwarzenberg et al., 2024). The selected safety factors were 100, 150, 175, and 250 times the physiological bending moment of 6.78 Nmm (Schwarzenberg et al., 2024) (Table 1). These safety factors provided a clinically relevant anchor point to describe the upper bound of survival and the limit of consistent failure.

**TABLE 1:** Loads used during the biomechanical tests of the four groups.

Group [-]	Peak force [N]	Bending moment [Nmm]	Safety factor [-]	Quantity [-]
1	93.5	678	100	10
2	140.3	1,017	150	10
3	163.7	1,187	175	10
4	233.8	1,695	250	10

### 2.2 Surgical procedures

The bones were placed into their respective custom 3D printed cutting guides and pilot holes were drilled through the cortices with a 1.1 mm diameter surgical drill bit (DePuy Synthes, Zuchwill, Switzerland). Through the cutting slot of the cutting guide, an osteotomy was performed with a 0.6 mm thick oscillating saw blade (DePuy Synthes). Additionally, 1.8 mm pilot holes were drilled into the distal and proximal epiphyses of the bones to insert anchorage screws required for embedding the samples.

Following cutting and drilling, an outline for the planned AdhFix osteosynthesis was drawn onto the dorsal side of the sample with a surgical marker. The desired dimensions of the AdhFix patch were 6 mm in width and 25 mm in length, extending 3 mm from either side of the pilot holes medially and laterally and 2.5 mm longitudinally from either end of the distal and proximal pilot holes. Stainless-steel cortical screws (diameter: 1.5 mm, length: 25 mm, DePuy Synthes) were cut to unicortical length as measured by a depth gauge before being inserted into the bone, ensuring that they would not pass through or contact the opposite cortex. A screwdriver was then used to partially insert the cortical screws into the pilot holes.

Using the method outlined in Hutchinson et al., the AdhFix composite (Bonevolent™ AdhFix, Biomedical Bonding AB, Stockholm, Sweden) was applied to the bone sample within the rectangular outline previously drawn (Hutchinson et al., 2021). First, the two cortical screws on one bone fragment were surrounded by the composite and then tightened with a screwdriver until they were “two-finger tight.” The composite was then cured with two 5 s light pulses from a high-energy visible light source (Bluephase PowerCure LED lamp, Ivoclar Vivadent Clinical, Schaan, Liechtenstein). The 0.8 cm diameter light source provided 2000 mW/cm^2^ and curing was repeated to cover the entire area of the composite. This process was repeated on the other bone fragment. Next, the two fragments were reduced using surgical clamps and the AdhFix composite was applied and cured across the osteotomy to bridge the two bone fragments. Finally, the entire 25 mm × 6 mm osteosynthesis surface was covered with a layer of the AdhFix composite and cured. When fabricating the AdhFix patches, the aim was to produce patches with a mean height between 1.5 mm and 2.0 mm.

### 2.3 Mechanical testing

Before mechanical testing, the distal and proximal epiphyses of the bone sample were embedded in blocks of polymethyl methacrylate (PMMA) which acted as the contact points for the four-point bending fixture used for the mechanical tests. First, 3 × 12 mm wood screws were inserted into the distal and proximal epiphyses to anchor the bone sample in the PMMA blocks. Custom Teflon (PTFE) molds were used to embed and align the distal and proximal bone epiphyses in PMMA to create 30 mm × 30 mm x 20 mm blocks, as shown in Figure 1c.

**FIGURE 2:**
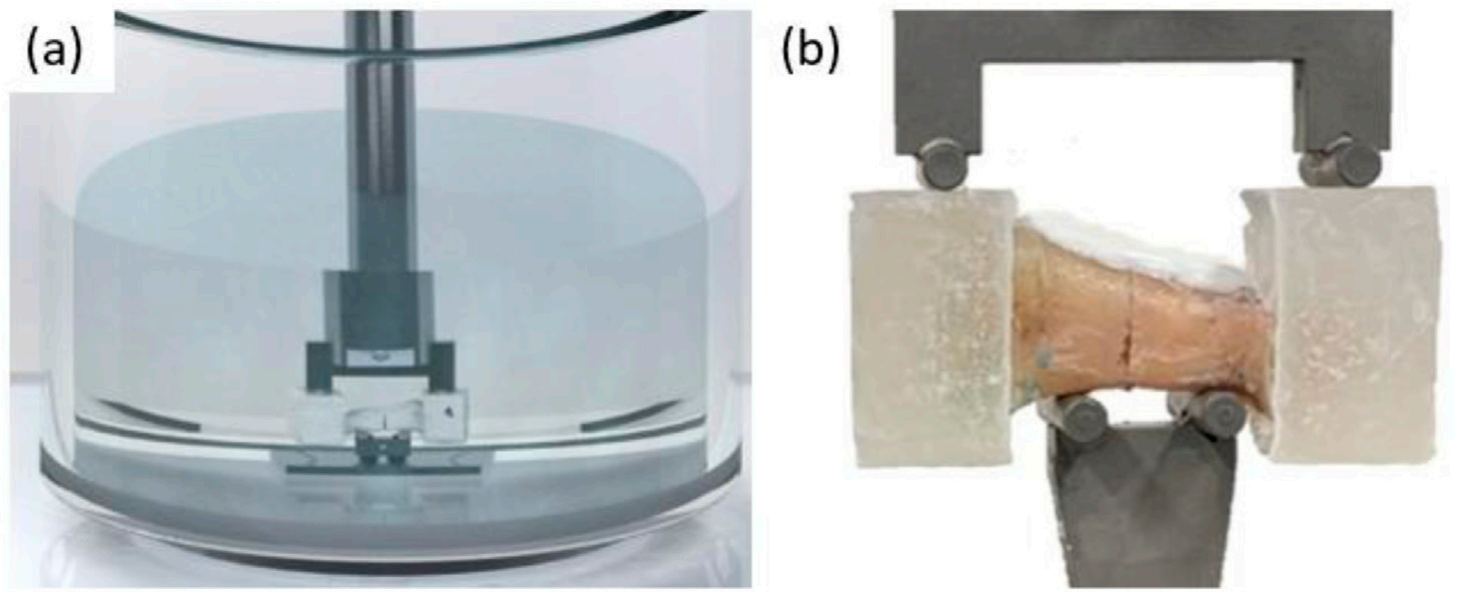
**(a)** SolidWorks rendering of experimental setup with the sample submerged in Ringer solution and the machine actuator. **(b)** Four-point bending fixture with a representative sample.

The mechanical testing was performed with an electromechanical testing system (Acumen III, MTS Systems Corporation, Eden Prairie, MN, USA) to apply cyclic four-point bending to the bone samples in a near-physiological environment. The applied sinusoidal force signal varied between a valley load of 2 N and a peak load which was altered depending on the testing group. The frequency was fixed at 1 Hz. For thirty minutes prior to the start of the test and throughout the testing which lasted at most 3.5 h, the samples were submerged in a bath of Ringer solution heated to 37°C. Figure 2 shows (a) a SolidWorks rendering of the experimental testing set up with the sample submerged in Ringer solution and the machine actuator and (b) a representative sample placed on the four-point bending fixture. The temperature was recorded continuously throughout the test with a thermometer probe (RTD temperature probe, Jumo Ltd., Fulda, Germany). The stop criterion in the test protocol was 12,600 cycles or failure of the sample, whichever occurred first. Sample failure was defined as a displacement limit set at −1.5 mm. This displacement limit was determined empirically through pilot testing and represents catastrophic failure of the specimen. At the start of each test, the testing system was operated in displacement-control mode until contact with the sample was established. Contact was established by lowering the piston at 1 mm/s until a force of 5 N was achieved, then returning to the valley load with a force of 2 N. At this point, the displacement was zeroed, and the cyclic force application began. The axial displacement, axial force, number of cycles, and temperature were recorded continuously with a sampling frequency of 100 Hz throughout the tests.

### 2.4 Post-test processing

The failure type was classified as composite failure over the fracture line, at the screw head, or by delamination from the bone surface. Digital calipers were used to measure the width of the AdhFix patch at the location of each screw. If the sample fractured, the widths and heights at both the proximal and distal side of the fracture were measured. If the sample did not fracture, it was fractured by hand at the osteotomy line to perform these measurements.

The data analysis was performed with a Python (v3.11) script to calculate the variables of interest for each sample, notably, the number of cycles to failure and the status (failed vs. survived). The number of cycles to failure were rounded up to the nearest cycle. Therefore, any monotonic failures had one cycle to failure. Additionally, the mean temperature across the test, the displacement amplitude in the last cycle, the mean patch width, and the mean patch height were summarized for each sample.

### 2.5 Statistical analysis

The statistical analysis was performed using R (version 4.3.1). Kaplan-Meier survival curves were plotted for each testing group depicting the survival probability as a function of the number of cycles. A log-rank test was calculated to determine if there was a statistically significant difference between the survival curves of the different testing groups. A one-way ANOVA with a *post hoc* analysis using a Bonferroni adjustment was calculated and displayed with the corresponding box plots to discern the statistical difference among the displacement amplitudes within the last cycle of the different testing groups. Unpaired t-tests were performed and portrayed with box plots of the mean patch height and of the displacement amplitude of the last cycle of the samples that failed compared to those that survived. Statistical significance was considered with a p-value of less than 0.05.

## 3 Results

**FIGURE 3:**
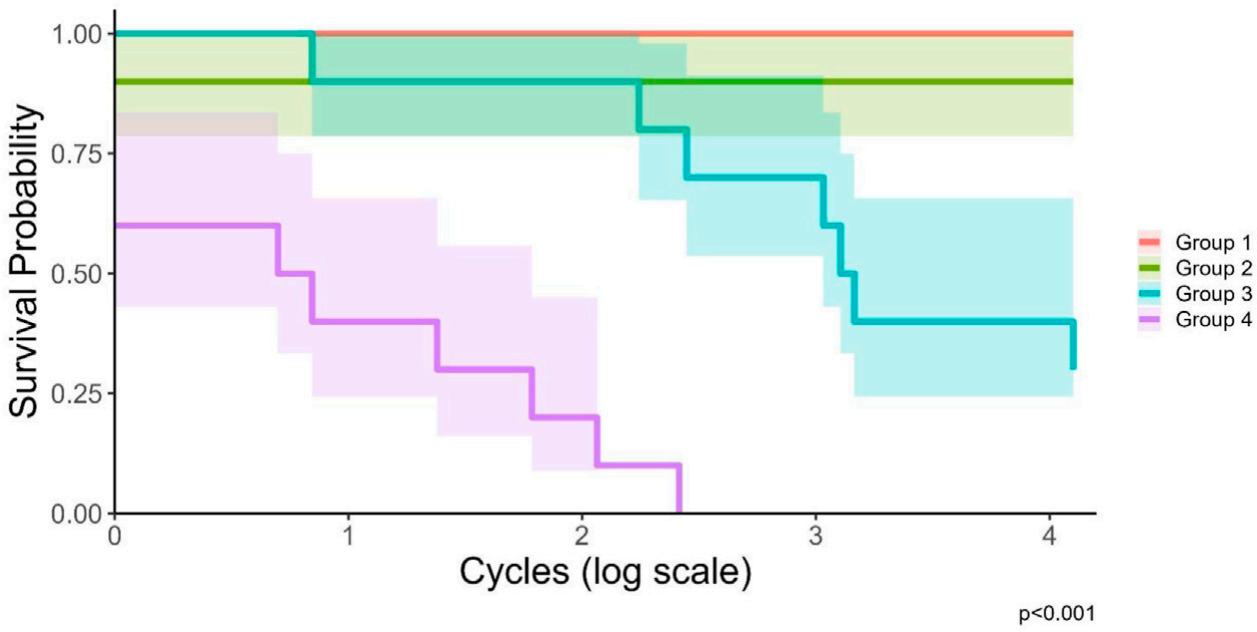
Kaplan-Meier survival curves for each testing group with 95% confidence intervals, displayed on a log-scale. The p-value of <0.001 displays the results of the log-rank test indicating a statistically significant difference between the survival curves of the different testing groups.

All samples survived at the lowest peak force level (Group 1, 93.5N), whereas all failed at the highest peak force level (Group 4, 233.8N) (Table 2; Figure 3). The two groups at intermediate peak force levels had a mixture of failures and survivors. Out of the 19 failed samples, 10 failed at a screw head, 5 patches delaminated from the bone surface, and 3 failed at the fracture line. The survival curves in Figure 3 display the survival probability as a function of the cycle count for each of the four testing groups. The log-rank test performed to compare these survival curves indicated that the survival curves were significantly different (Χ^2^ (3, N = 40) = 47.9, p < 0.001). Pairwise comparison further showed that Group 1 and Group 2 were not significantly different from each other (p = 0.3) while all other group combinations were significantly different from each other (p ≤ 0.01).

**TABLE 2:** Failure number and mode for each testing group (each group has an N = 10).

Group [-]	Number failed [-]	Monotonic failures [-]	Failure at a screw head	Failure by delamination	Failure at the fracture line
1	0	0	0	0	0
2	1	1	0	0	1
3	7	0	5	2	0
4	10	4	5	3	2

**FIGURE 4:**
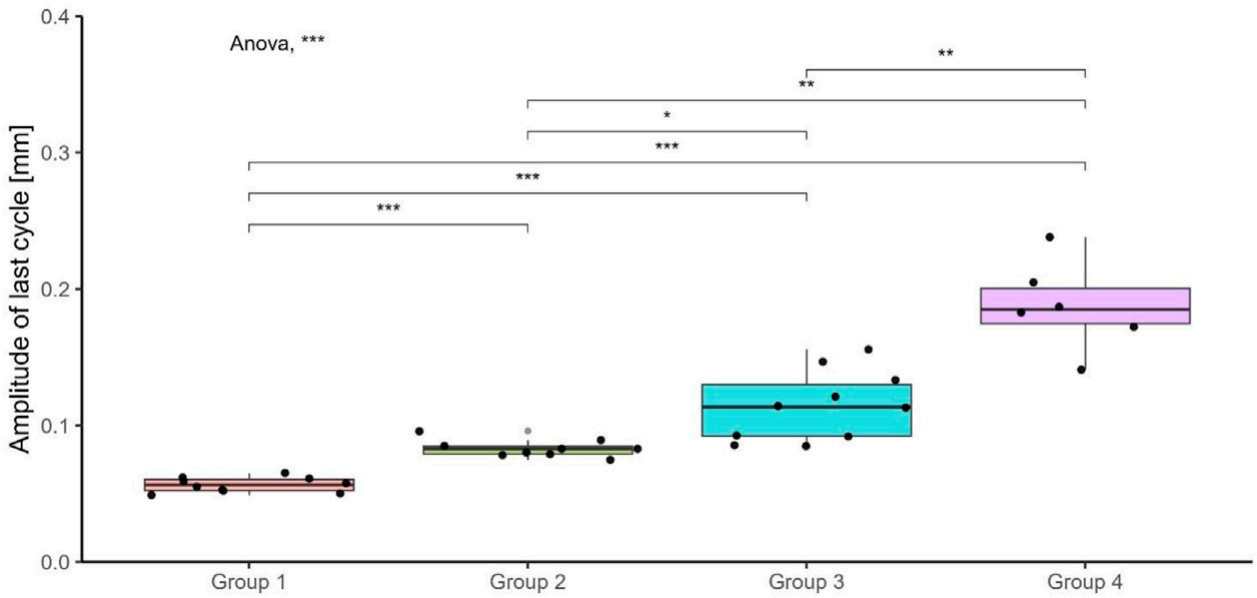
Boxplots of the displacement amplitude of the last cycle for the different testing groups. The samples which failed monotonically are not presented in this plot. The p-values indicate the results of a one-way ANOVA with *post hoc* analysis for which unpaired, Bonferroni-adjusted t-tests were calculated to statistically compare the amplitude between the groups. ns: non-significant, *: p ≤ 0.05, **: p ≤ 0.01, ***: p ≤ 0.001.

**FIGURE 5:**
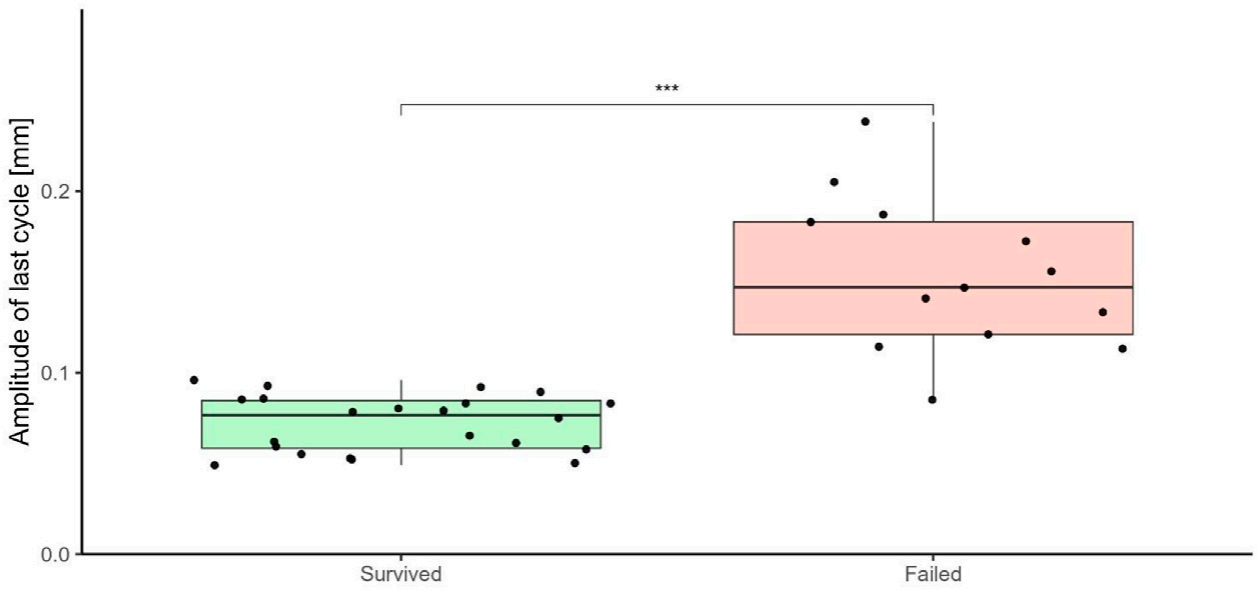
Boxplots of the displacement amplitude of the last cycle, comparing the samples that failed with those that survived. The p-value indicates the results of an unpaired t-test to statistically compare the displacement amplitude of the last cycle between the two groups. ns: non-significant, *: p ≤ 0.05, **: p ≤ 0.01, ***: p ≤ 0.001.

The displacement amplitude in the last cycle was also significantly different between the testing groups (Figure 4). The mean displacement amplitude of the last cycle was found to increase with increasing peak force. The last cycle’s displacement amplitude was also significantly different between the samples that failed and those that survived (p < 0.001, Figure 5). The samples that failed had a mean displacement amplitude in the last cycle of 0.15 mm (standard deviation (SD) = 0.043 mm) (not considering the monotonic failures), whereas those that survived had a mean of 0.072 mm (SD = 0.016 mm).

The dimensions of the AdhFix patches were measured with a width of 7.06 mm (SD = 0.58 mm) and a height of 1.61 mm (SD = 0.25 mm, range: 1.14–2.19 mm). The patch heights were not statistically significantly different between the failed and survived samples (p = 0.113).

## 4 Discussion

This study provided insight into the fatigue behavior of the AdhFix fixation technique tested on well-reduced ovine phalanx fractures in controlled near-physiological conditions. The key outcome of this study was the development of experimental and analysis methods for fatigue testing of the AdhFix osteosynthesis that was deemed more challenging than regular standard metal implants. Additionally, the bounds of failure and survival were explored for a loading cycle number representing a typical rehabilitation protocol for hand fracture treatment. These results allow for an awareness of the limits of this osteosynthesis technique in cyclic testing. While this study focused on fully reduced ovine proximal phalanx fractures, these methods could be extended to other anatomies, loading modalities, and fracture types.

In this study, it was observed that the type of failure changed as the force increased for the four testing groups. The testing group with the highest peak force experienced the most monotonic failures. The monotonic failures represent failures due to overloading of the specimen, whereas fatigue failures represent failures due to a gradual decrease in the mechanical properties due to cyclic loading (Kelly et al., 2017). Fatigue is a stochastic process governed by probability functions, meaning that at any given cycle there is an unknown probability that failure can occur (Shen et al., 2000). From analyzing the survival curves, presented in Figure 3, it is possible to see that at higher peak forces the number of cycles to failure was lower on average than the number of cycles to failure at the lower peak forces. Therefore, two trends can be observed from the results: as failure occurrence increases, the likelihood of monotonic failures also increases, and as the peak force increases, the number of cycles to failure decreases. From this rationale, the monotonic failure in Group 2 can be explained, where otherwise, Groups 1 and 2 were identical. Understanding how the samples fail is also of importance during the development of a new osteosynthesis technique. The patches failed in different ways, but it was never the case that the bone itself failed. When analyzing the failure types, it was observed that all failures due to fracture over the osteotomy were monotonic failures. However, not all monotonic failures failed at the osteotomy, with one failing at a screw head and another failing due to a delamination of the AdhFix osteosynthesis from the bone surface. Interestingly, one sample failed after the end of a test when the application of force was removed. It is highly probable that this sample had already broken before the end of the test but was being held in place by traction or frictional forces. This elucidates another failure mechanism that could not be adequately explored in this study.

Previous monotonic testing using the same four-point bending configuration as in the current study, but without cyclic loading or the bioreactor, showed that AdhFix constructs were stiffer than traditional metal plates in well-reduced fractures (Schwarzenberg et al., 2023). In contrast, AdhFix was less stiff in comminuted fracture models. Despite these differences in stiffness, both AdhFix groups demonstrated significantly lower maximum bending moments to failure compared to metal implants. A subsequent study placed these results in a clinical context by demonstrating that the forces encountered during early rehabilitation are substantially lower than the failure thresholds observed in the AdhFix constructs (Schwarzenberg et al., 2024). This suggests that even in cases where mechanical strength is lower, the fixation may still be adequate under realistic loading conditions during the rehabilitation period. When inferring performance under cyclic loading based on these previous monotonic results, it is reasonable to expect that metal fixation systems may offer higher mechanical safety margins, particularly in situations where load demands are elevated. It is also important to note that the failure behavior differed between materials. Metal implants typically failed catastrophically, often resulting in bone damage, whereas AdhFix constructs failed within the patch material itself. This distinction may be clinically meaningful, as patch-level failure could preserve bone integrity and simplify any necessary revision procedures. Given the higher failure loads observed in monotonic tests, it is likely that metal constructs would demonstrate superior performance under cyclic loading. Typically, when studying the fatigue behavior of a given material, S-N curves are used which relate the applied stress to number of cycles to failure (Kelly et al., 2017). The fatigue behavior of metals has been extensively researched and there are well-established relationships between stress and number of cycles to failure for various metals (Kelly et al., 2017). This method of analysis was untenable for this study because of the high degree of variability in the number of cycles to failure for samples with the same peak force. Instead, survival curves were employed to allow for a statistical comparison of the testing groups surpassing a binary categorization of survivors and failures and considering the difference in the number of cycles to failure. The log-rank test applied to the survival curves indicated a statistically significant difference between the testing groups, expressing a dependency of failure and number of cycles to failure on the peak force. Survival curves are customarily used in clinical research but can also be applied to engineering constructs, and in engineering, this method is often termed “reliability analysis” (Zhanshan Ma, 2008).

An analysis of the displacement amplitude of the last cycle allows for a quantitative assessment of the cyclic behavior of the osteosynthesis technique. The displacement amplitude is related to the material behavior of the AdhFix patch and bone construct. The elastic strain is recovered during the unloading part of each cycle, whereas the plastic strain is not recoverable (Peellage et al., 2023). For materials which exhibit viscoelastic plastic behavior, the displacement amplitude will become progressively smaller with each cycle as the viscoelastic behavior dominates the response (Peellage et al., 2023). It is critical to note that the displacement amplitude for the last cycle was calculated for the last cycle of each individual sample which varies for the samples that failed cyclically and was always at 12,600 cycles for the samples that survived. However, despite the difference in number of cycles to failure, the displacement amplitude nevertheless provides a quantitative measure of fixation stability and evidence that the cyclic deformation changes with increasing peak force. The displacement amplitude of the last cycle was also measured in previous studies investigating the mechanical properties of the AdhFix technique with the purpose of investigating the fixation stability compared to traditional fixation methods such as K-wires or metal plates where it was found to have superior or equal performance (Granskog et al., 2018; Hutchinson et al., 2021).

The goal was to fabricate patches with a mean height between 1.5 and 2.0 mm. In the study, the actual range of the height was much wider at 1.14-2.19 mm; however, the mean height (standard deviation) was 1.61 (0.25) mm which is within the target range. This widening of the targeted height range highlights the difficulty in reproducing identical patches each time. The results revealed that the AdhFix patch height was not significantly different between the samples that failed and those that survived. This was the desired outcome because in clinical practice, all patches should be capable of providing adequate strength and stability to the healing bone despite minor differences in patch height.

A previous study focused on determining the internal loads experienced by an osteosynthesis on a human proximal phalanx fracture under non-load bearing rehabilitation exercises (Schwarzenberg et al., 2024). This investigation established that the maximum internal bending moment in the osteosynthesis was 6.78 ± 1.62 Nmm. In the present study, the force levels determined from pilot testing were then rounded to match certain safety factors of this physiological bending moment. The samples were tested at safety factors between 100 and 250 times the physiological bending moment. While the internal loads study was a critical study for developing osteosynthesis techniques for proximal phalanges and presented methods which could be extended to other fracture sites in the human body, it is important to note that it was performed in a very controlled environment. In daily living, patients may experience much higher forces and moments due to non-compliancy to the prescribed rehabilitation guidelines, accidents, and combined loading scenarios that were not investigated. It is paramount that the osteosynthesis does not fail under any condition, and especially the non-ideal conditions of daily living. Therefore, the chosen peak forces were based on these considerations and on the results of the pilot testing. The development of an osteosynthesis is an iterative process and when investigating physiological loading, it is logical to begin with idealized, controlled conditions. The internal loads in non-compliant cases, in accidents, or in combined loading scenarios are also worth inspecting, but currently they remain unknown.

The primary limitation of this study was the high degree of variability in the fixation patches. One of the benefits of the AdhFix technique is *in-situ* customizability. However, the customizability presents itself as a disadvantage during mechanical evaluations because it is impossible to create two identical patches. All patches differed in their width and height, both when comparing different points within a single sample and comparing between samples. These differences are a result of intra-operator variability as a single operator performed all fixations in this study. Additionally, increased patch thickness resulting from variability in the application of the light-curable composite could potentially interfere with postoperative tendon movement. However, determining the critical threshold of patch thickness that significantly impacts clinical outcomes requires further targeted investigations beyond the scope of this initial biomechanical evaluation. The intra-operator variability was further exacerbated by the fact that the patches were produced on different days across several weeks of testing. However, a previous study investigating this showed intra-operator effect does not play a large role mechanically (Colding-Rasmussen et al., 2023). Additionally, no two bone samples were identical. Slight changes in the bone geometry or osteotomy could influence the patch construction and therefore the overall results of the biomechanical tests. While this study implemented ovine phalanges, porcine metacarpals have been used in similar studies (Hutchinson et al., 2021). The next limitation is associated with the reduction of the osteotomy. In more than half of the samples, a very small gap in the volar cortex was observed after reducing the fracture and fixating it with the AdhFix patch. The gaps were of varying sizes, however unmeasurable with the digital calipers. This represents another source of variability in the study. During the cyclic tests, it is likely that the first unknown number of cycles involved a closing of the gap, until a full reduction was achieved. Compared to a perfectly reduced sample with no noticeable gap, the mechanical behavior, especially at the beginning of the test, may have been altered. Nevertheless, it is difficult to hypothesize about how these gaps may have affected the results, if at all. In clinical practice, however, these gaps could also present themselves and would be much more difficult to perceive and control. Additionally, comminuted fractures or fractures involving joint surfaces were beyond the scope of this experimental design, and their mechanical behavior under multi-axial loading conditions presents additional complexity not assessed by cyclic four-point bending alone. Future studies would need to incorporate more sophisticated mechanical testing setups and multi-directional loading conditions to fully evaluate the performance and suitability of the AdhFix system in these more clinically challenging fracture scenarios.

This study represents the first time the AdhFix patches were tested cyclically in near-physiological conditions with a clinically relevant number of cycles. Previous cyclic testing on these osteosyntheses were performed to 1,000 cycles and in dry conditions (Hutchinson et al., 2021). These previous tests also did not investigate the characteristics of failure as all samples survived and only one peak load of 70 N was used (Hutchinson et al., 2021). It is important to establish the occurrence of fatigue failure and understand how these failures occur. This present study also considered the physiological bending moment present in a proximal phalanx osteosynthesis with knowledge from a recently published study (Schwarzenberg et al., 2024). Additionally, this present investigation provides an extension of previous work on monotonic failures, by using the same four-point bending fixtures with the addition of cyclic testing and the Ringer solution heated to 37°C.

In summary, the AdhFix osteosynthesis technique was applied to fixate well-reduced ovine proximal phalanx fractures. The constructs were then tested cyclically in physiological conditions, and the patches demonstrated a dependency on the peak force of the cyclic testing, with all samples surviving at the lowest peak force (100x the expected loading) and all failing at the highest peak force (250x the expected loading). This study provides a framework for testing novel osteosynthesis devices cyclically and advances the understanding of the fatigue behavior of the AdhFix osteosynthesis technique and exposes its limits.

## Data Availability

The datasets presented in this study can be found in an online repository. The name of the repository and accession number can be found below: https://doi.org/10.5281/zenodo.13843571.
